# Physical Activity, Sitting Time, and Mortality From Inflammatory Diseases in Older Adults

**DOI:** 10.3389/fphys.2018.00898

**Published:** 2018-07-12

**Authors:** Verónica Cabanas-Sánchez, Pilar Guallar-Castillón, Sara Higueras-Fresnillo, Esther García-Esquinas, Fernando Rodríguez-Artalejo, David Martinez-Gomez

**Affiliations:** ^1^Department of Physical Education, Sport and Human Movement, Autonomous University of Madrid, Madrid, Spain; ^2^Department of Preventive Medicine and Public Health, School of Medicine, Universidad Autónoma de Madrid, IdiPaz, Madrid, Spain; ^3^CIBERESP and IMDEA-Food Institute, CEI UAM+CSIC, Madrid, Spain; ^4^IMDEA-Food Institute, CEI UAM+CSIC, Madrid, Spain

**Keywords:** physical activity, sitting time, mortality, inflammation diseases, infectious diseases

## Abstract

**Objective:** The aim of this study was to examine the independent and combined associations of physical activity (PA) and sitting time (ST) with long-term mortality attributed to inflammatory causes other than cardiovascular disease (CVD) and cancer in a national cohort of older adults in Spain.

**Design:** Prospective study.

**Setting and Participants:** A cohort of 3,677 individuals (1,626 men) aged ≥60 years was followed-up during 14.3 years.

**Measures:** At baseline, individuals reported PA and ST. The study outcome was death from inflammatory diseases when CVD or cancer mortality was excluded. This outcome was classified into infectious and non-infectious conditions. Analyses were performed with Cox regression and adjusted for PA, ST, and other main confounders (age, sex, educational level, smoking, alcohol consumption, body mass index, and chronic conditions).

**Results:** During follow-up, 286 deaths from inflammatory diseases (77 from infectious diseases) were identified. Compared to individuals who defined themselves as inactive/less active, mortality from inflammatory diseases was lower in those who were moderately active (hazard ratio [HR] = 0.67, 95% confidence interval [CI] = 0.50–0.90) or very active (HR = 0.48, 95%CI = 0.33–0.68), independently of ST. Also, being seated ≥7 h/d vs. <7 h/d was linked to higher mortality (HR = 1.38, 95%CI = 1.02–1.87). The largest risk of mortality was observed in inactive/less active individuals with ST≥7 h/d (HR = 2.29, 95%CI = 1.59–3.29) compared to those with moderate/very PA and ST <7 h/d. Low PA and high ST were consistently associated with a higher risk of mortality from non-infectious inflammatory causes. Associations of PA and ST with mortality from infectious inflammatory causes showed a similar trend, but most of them did not reach statistical significance.

**Conclusions:** Low PA and high ST were independently associated with higher mortality from inflammatory diseases other than CVD or cancer in older adults. Interventions addressing simultaneously both behaviors could have greater benefits than those focusing on only one of them.

## Introduction

Inflammation is a physiological response to tissue injuries or infections (Addison et al., 2012). However, when inflammation persists, chronically elevated systemic levels of pro-inflammatory cytokines can lead an increased risk of inflammatory diseases. In this sense, chronic inflammation is known to play a central role in the etiology of cardiovascular disease (CVD) and cancer (Singh-Manoux et al., 2017) and plays a major pathogenic role in other diseases such as diabetes, chronic kidney disease, inflammatory bowel, cirrhosis, chronic obstructive pulmonary disease, rheumatoid arthritis, and several neurodegenerative conditions (Dungey et al., 2013; Holmes, 2013; Wang et al., 2013).

Aging is a progressive process associated with several physiological changes, chronic inflammation and a deterioration in homeostatic functions, which contributes to increased oxidative stress (Salminen et al., 2012). Indeed, compared with young or middle-age adults, older populations have an increased incidence of infectious and other inflammatory conditions (Bender, 2003).

There is evidence that regular physical activity (PA) produces anti-inflammatory actions that might prevent cardiovascular disease (CVD), cancer and many other age-associated conditions, including mitochondrial dysfunction, frailty, sarcopenia, and physical and mental disability (Chen et al., 2014; Sallam and Laher, 2016). By contrast, sedentary behavior, defined as any waking behavior characterized by low levels of energy expenditure, is linked to low-grade inflammation, independently of PA or adiposity (Henson et al., 2013). Although the effect of PA and sitting time (ST) on CVD and cancer mortality has been widely studied in adults (Nocon et al., 2008; Katzmarzyk et al., 2009; Wilmot et al., 2012), no previous investigation has focused on mortality from other diseases with a major inflammatory component in older adults. Accordingly, this work examined the independent and combined association of PA and ST with long-term mortality from inflammatory diseases, after excluding CVD or cancer mortality, in a national cohort of older adults.

## Methods

### Study design and participants

We analyzed data from the *Universidad Autónoma de Madrid* (UAM) cohort with a total of 4,008 individuals (1,739 men) representative of the non-institutionalized population aged 60 years and older in Spain. The study methods have been reported elsewhere (León-Muñoz et al., 2013; Martínez-Gómez et al., 2015). In short, participants were recruited between October 2000 and February 2001 using probabilistic sampling by multistage clusters. The clusters were stratified according to region of residence and size of municipality. Then, census sections and households were chosen randomly within each cluster. Finally, study participants were selected from 420 census sections in sex and age (60–69, 70–79, and ≥80 years) strata. The information was collected through home-based personal interviews using a structured questionnaire, followed by a physical examination performed by trained and certified personnel. The study response rate was 71%. The study was approved by the Clinical Research Ethics Committee of *La Paz* University Hospital (Madrid, Spain), and written informed consent was obtained from all study participants and an accompanying family member.

### Physical activity and sitting time

Information on PA and ST was self-reported. PA was obtained with a global question that asked participants to rate their level of PA in comparison with their age-peers in four categories: inactive, less active, moderately active, and very active (Martínez-Gómez et al., 2015). Because the prevalence of the lowest PA category was 7.21%, those belonging to the categories “inactive” and “less active” were merged into the same one.

ST was estimated by leisure time spent sitting down based on the following question (León-Muñoz et al., 2013): “*About how much time per day do you spend sitting down on weekdays? Please add up the total number of hours that you spent sitting down regardless of the activity that you do (eating, listening to the radio, watching television, reading, sewing, driving, etc*.).” The same question was asked with reference to weekend days. The number of hours per day was calculated as follows: [(weekday STx5 + weekend day STx2)/7]. ST was classified into tertiles with cut-points at 3.29 and 5.29 h/d.

### Mortality

All-cause deaths among study participants, from study baseline at 2000/2001 to the end of follow-up at 31 December 2015, were identified by a computerized search of the National Death Index, which contains information on the vital status of all residents in Spain. The vital status was ascertained for 99.9% of the cohort. We considered inflammatory diseases other than CVD or cancer as those where inflammation or infection play a major pathogenic role; these diseases can be further classified as infectious and non-infectious. In accordance with previous studies (Andersen et al., 2006), we selected deaths with the following codes of the tenth revision of the International Classification of Diseases (ICD-10): (a) Infectious diseases (A04.7, A41.9, B18.2, J18, J18.0, J18.1, J18.9, J22, and N39.0) and (b) non-infectious diseases: chronic neurodegenerative diseases (G20, G30.1, and G30.9), diabetes (E11.9, E14.5, E14.7, and E14.9), chronic lower respiratory diseases (J40, J42, J43.9, J44, J44.1, J44.8, J44.9, J45.9, and J47), cirrhosis (K74.6), kidney failure (N12, N18.9, and N19), other diseases of the respiratory system (J69, J69.0, and J84.1), cholecystitis (K81.9), and other diseases (E85.4 and N32.1).

### Covariates

Age and sex were recorded. Educational level was evaluated as the highest level achieved (no formal education, primary, and secondary or higher). Participants also reported whether they were never, former, or current smokers. Alcohol consumption was obtained with the frequency-quantity scale used in the Spanish National Health Survey (Guallar-Castillón et al., 2001). Firstly, individuals rated their alcoholic beverage consumption among the following options: abstainer, former drinker, and current/sporadic drinker. Then, those who indicated current/sporadic drinking also reported the frequency and quantity of beer, wine, and spirits consumed during the past year. Total alcohol intake was classified into excessive and moderate consumption using cutoff points of >30 g/d in men and >20 g/d in women (Chalasani et al., 2012). Weight and height were measured using standardized procedures (Gutiérrez-Fisac et al., 2004), and the body mass index (BMI) was calculated as weight in kilograms divided by height in meters squared. Finally, information on the following chronic conditions diagnosed by a physician and reported by the study participants was recorded: chronic lung disease, cardiovascular disease, diabetes mellitus, Parkinson, and cancer at any site.

### Statistical analyses

Of the 4,008 participants, 331 were excluded because of missing data on one or more of the study variables. Thus, the analyses were conducted with 3,677 individuals (1,626 men). We initially compared risk estimates for sex and age (<70 and ≥70 years) strata and no significant interactions were observed (all *p* > 0.05); therefore, the analyses were performed for the total sample. Baseline characteristics of the study sample by categories of PA and ST were presented as mean ± SD or %. Cramer's V was used to describe the relationship between groups of PA and ST.

The association of PA (inactive/less active, moderately active, and very active) and ST (tertiles) with mortality from inflammatory diseases was summarized with hazard ratios (HR) and their 95% confidence intervals (CI) obtained from Cox regression. Three models with progressive adjustment for potential confounders were fitted. The first model adjusted for age and sex; the second model further adjusted for the rest of potential covariates; and the third model also included PA or ST (as appropriate) to examine the independent association of both behaviors. The P for trend was calculated by modeling the categories of PA and the tertiles of ST as continuous variables. Moreover, to assess the dose-response relationship between inflammatory mortality and ST as a continuous variable, we fitted a restricted cubic spline, with adjustment as in model 3, testing the departure from a linear trend.

To examine the combined effect of PA and ST on inflammatory mortality, first, we considered as health risk behaviors (HRBs) the categories of PA (inactive/less active) and ST (≥7 h/d) showing a detrimental role on inflammatory mortality in the aforementioned individual associations. Then, we created four categories of health risk behaviors: (1) 0 HRBs: high PA and low ST; (2) 1 HRB (high ST): high PA and high ST; (3) 1 HRB (low PA): low PA and low ST; and (4) 2 HRBs: low PA and high ST. Analyses using those categories were performed with Cox regression and adjusted as in models 1 and 2.

To rule out the effect of subclinical disease on the study results and to reduce the likelihood of reverse causation, we replicated the analyses after excluding 200 individuals who died during the two first years of follow-up.

We assessed the assumption of the proportionality of mortality hazards both graphically and by testing the significance of interaction terms for the main exposure variables and years of follow-up. No evidence was found of departure from the proportional hazards assumption (*P* > 0.05).

Analyses were performed with STATA® v.14.0, and statistical significance was set at *P* < 0.05.

## Results

The main characteristics of the study participants at baseline are shown in Table 1. Differences in all variables were identified among the PA level groups (all *P* < 0.01). Compared to those in the lowest tertile of ST, those in the highest tertile were older, showed lower educational level, were more likely to be never drinkers, had higher BMI, and had suffered more frequently from coronary heart disease or diabetes (all *P* < 0.05). PA and ST were weakly associated (*Cramer's V* = 0.245, *P* < 0.05). Interaction between PA and ST was close to significance (i.e., *P* = 0.063 for inflammatory causes mortality).

**Table 1:** Characteristics of study participants at baseline.

	**All**	**Physical activity**				**Sitting time**			
		**Inactive/less active**	**Moderately active**	**Very active**	***P***	**Tertile 1 (lowest)**	**Tertile 2**	**Tertile 3 (highest)**	***P***
*N*	3,677	920	1,576	1,181	–	1,297	1,181	1,199	–
Age (years), mean ± *SD*	71.72 ± 7.90	74.77 ± 8.26	71.10 ± 7.66	70.17 ± 7.28	<0.001	70.01 ± 7.34	71.13 ± 7.47	74.15 ± 8.31	<0.001
Men, %	44.22	37.15	43.98	50.04	<0.001	44.55	45.31	42.79	0.347
**EDUCATIONAL LEVEL, %**									
No education	51.84	61.55	51.56	44.65	<0.001	48.29	49.11	58.35	<0.001
Primary	34.79	28.83	34.85	39.34		38.88	36.24	28.93	
Secondary or higher	13.38	9.61	13.60	16.01		12.83	14.65	12.72	
**SMOKING STATUS, %**									
Never	65.34	67.99	66.26	62.07	0.001	66.25	64.93	64.78	0.627
Former	24.33	23.90	23.96	25.15		24.11	23.47	25.40	
Current	10.33	8.10	9.79	12.79		9.64	11.60	9.83	
**ALCOHOL CONSUMPTION, %**									
Never	49.32	55.83	51.12	41.84	<0.001	42.77	51.14	54.61	<0.001
Former	11.92	16.21	11.10	9.67		9.96	11.06	14.88	
Moderate^I^	28.59	21.73	27.39	35.52		35.87	27.22	22.06	
Heavy^I^	10.18	6.22	10.39	12.97		11.40	10.59	8.45	
Body mass index (kg/m^2^), mean ± *SD*	28.84 ± 4.60	29.43 ± 5.39	28.90 ± 4.42	28.30 ± 4.07	<0.001	28.71 ± 4.34	28.73 ± 4.34	29.10 ± 5.09	0.034
Chronic lung disease, %	14.09	21.05	12.81	10.39	<0.001	12.07	12.15	18.20	0.089
Coronary heart disease, %	8.61	14.50	7.47	5.54	<0.001	5.79	8.87	11.40	<0.001
Diabetes mellitus, %	15.29	20.02	15.40	11.46	<0.001	12.70	15.32	18.06	0.011
Parkinson, %	1.46	2.87	0.92	1.08	<0.001	1.10	0.90	2.40	0.075
Cancer, %	1.79	3.11	1.11	1.66	0.006	1.60	1.37	2.40	0.908
**PHYSICAL ACTIVITY, %**									
Inactive/low active	25.01	–	–	–	–	14.66	15.81	45.26	<0.001
Moderately active	42.87	–	–	–		42.37	49.53	36.84	
Very active	32.12	–	–	–		42.97	34.66	17.89	
Sitting time (h/d), mean ± *SD*	4.79 ± 2.74	6.48 ± 3.61	4.47 ± 2.08	3.91 ± 2.09	<0.001	2.39 ± 0.71	4.38 ± 0.52	7.80 ± 2.59	<0.001

I Threshold between moderate and heavy drinker: 10g^*^d^−1^ in women and 20 g^*^d^−1^ in men.

During a mean follow-up of 10.77 years (median = 13.73 years, range, 0.02–14.25), corresponding to 50,485 person-years, 1,669 (45.39%) deaths occurred. Among them, there were 286 inflammatory deaths other than CVD or cancer: 77 were from infectious diseases and 209 from non-infectious diseases (Supplementary Table 1).

A higher PA level was associated with a progressively lower risk of death from total inflammatory diseases (*P* < 0.001) (Table 2). In fully adjusted models, compared to inactive/less active individuals, the HR (95% CI) of mortality from inflammatory diseases was 0.67 (0.50–0.90) in those who were moderately active and 0.48 (0.33–0.68) in those who were very active. Results were similar for infectious and non-infectious diseases, but they achieved statistical significance only for non-infectious diseases.

**Table 2:** Mortality risk for inflammatory causes according to levels of physical activity and sitting time in older adults.

	**Physical activity (categories)**				**Sitting time (tertiles)**			
	**Inactive/less active**	**Moderately active**	**Very active**	**P for trend**	**Tertile 1 (lowest)**	**Tertile 2**	**Tertile 3 (highest)**	**P for trend**
*N*	920	1,576	1,181		1,297	1,181	1,199	
**INFLAMMATORY CAUSES (*****n*** = **286)**								
Deaths	110	117	59		77	82	127	
Model 1, HR (95% CI)	1.00 (Ref.)	0.61 (0.46–0.82)	0.41 (0.29–0.58)	<0.001	1.00 (Ref.)	1.12 (0.79–1.57)	1.61 (1.17–2.21)	0.003
Model 2, HR (95% CI)	1.00 (Ref.)	0.64 (0.47–0.86)	0.44 (0.31–0.63)	<0.001	1.00 (Ref.)	1.07 (0.75–1.53)	1.49 (1.08–2.06)	0.014
Model 3, HR (95% CI)	1.00 (Ref.)	0.67 (0.50–0.90)	0.48 (0.33–0.68)	<0.001	1.00 (Ref.)	1.06 (0.74–1.51)	1.28 (0.92–1.77)	0.140
**INFECTIOUS INFLAMMATORY CAUSES (*****n*** = **77)**								
Deaths	27	33	17		22	25	30	
Model 1, HR (95% CI)	1.00 (Ref.)	0.81 (0.48–1.37)	0.56 (0.30–1.04)	0.063	1.00 (Ref.)	1.19 (0.64–2.20)	1.22 (0.68–2.17)	0.507
Model 2, HR (95% CI)	1.00 (Ref.)	0.83 (0.49–1.40)	0.58 (0.31–1.09)	0.088	1.00 (Ref.)	1.20 (0.64–2.27)	1.22 (0.68–2.19)	0.509
Model 3, HR (95% CI)	1.00 (Ref.)	0.83 (0.49–1.41)	0.59 (0.31–1.13)	0.105	1.00 (Ref.)	1.20 (0.64–2.25)	1.12 (0.62–2.05)	0.712
**NON-INFECTIOUS INFLAMMATORY CAUSES (*****n*** = **209)**								
Deaths	83	84	42		55	57	97	
Model 1, HR (95% CI)	1.00 (Ref.)	0.55 (0.39–0.79)	0.36 (0.24–0.55)	<0.001	1.00 (Ref.)	1.09 (0.72–1.65)	1.78 (1.23–2.58)	0.002
Model 2, HR (95% CI)	1.00 (Ref.)	0.59 (0.41–0.84)	0.41 (0.27–0.62)	<0.001	1.00 (Ref.)	1.03 (0.67–1.58)	1.59 (1.08–2.33)	0.015
Model 3, HR (95% CI)	1.00 (Ref.)	0.63 (0.44–0.90)	0.45 (0.29–0.69)	<0.001	1.00 (Ref.)	1.01 (0.66–1.55)	1.33 (0.90–1.97)	0.139

*HR, Hazard ratio; CI, Confidence Interval. Cut points for sitting time tertiles were 3.29 and 5.29. Model 1 was adjusted for age (years) and sex (male/female). Model 2 was adjusted for age (years), sex (male/female), educational level (no formal studies/Primary studies/Secondary or higher studies), smoking (never smoker/ former smoker/current smoker), alcohol consumption (never drinker/former drinker/moderate drinker/heavy drinker), body mass index (kg/m^2^), chronic lung disease (no/yes), cardiovascular disease (no/yes), diabetes mellitus (no/yes), Parkinson (no/yes), and cancer (no/yes). Model 3 was adjusted as in Model 2 plus physical activity or sitting time (as appropriate)*.

**Figure 1:**
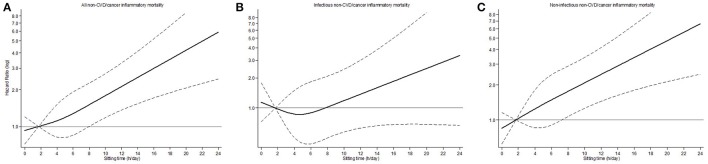
Hazard ratio and 95% confidence interval for mortality for inflammatory causes according to sitting time in older adults: **(A)** All-inflammatory causes. **(B)** Infectious inflammatory causes. **(C)** Non-infectious inflammatory causes. Footnotes: estimates are from Cox regression models of inflammatory, infectious inflammatory and non-infectious inflammatory causes mortality against a restricted cubic spline of sitting time with knots at 2, 4.14, and 8. The black line plots the hazard ratio, and the dashed lines indicate the upper and lower 95% confidence limits. Analyses were adjusted for age (years), sex (male/female), educational level (no formal studies/Primary studies/Secondary or higher studies), smoking (never smoker/ former smoker/current smoker), alcohol consumption (never drinker/former drinker/moderate drinker/heavy drinker), body mass index (kg/m^2^), chronic lung disease (no/yes), cardiovascular disease (no/yes), diabetes mellitus (no/yes), Parkinson (no/yes), cancer (no/yes), and physical activity (inactive or less active/moderately active/very active).

ST showed a direct dose-response relationship with mortality from inflammatory diseases, although it lost statistical significance after adjustment for PA (Table 2). In spline analyses (Figure 1), the association between ST and mortality showed a non-lineal trend (all *P* < 0.01), with mortality risks associated with ≥7 h/d in ST. This non-lineal trend was more evident for mortality attributed to infectious inflammatory causes. In fully adjusted models, compared to those with ST <7 h/d, participants with ST ≥7 h/d had a mortality HR (95%CI) of 1.38 (1.02–1.87) and 1.46 (1.02–2.10) for total and non-infectious inflammatory causes, respectively; the corresponding result for infectious diseases was 1.15 (0.68–1.97).

Table 3 shows the risk of mortality according to the number and type of HRBs. A HRB was defined as low PA (being inactive/less active) or high ST (≥7 h/d). The percentage of individuals with 0 HRBs, 1 HRB (high ST), 1 HRB (low PA), and 2 HRBs (high ST and low PA) was respectively 66.5, 8.4, 14.6, and 10.4%. In fully adjusted analyses, and compared with participants with 0 HRBs, the HR (95%CI) for mortality from inflammatory diseases was 1.45 (0.97–2.18) for individuals with 1 HRB (high ST), 1.68 (1.19–2.37) for individuals with 1 HRB (low PA), and 2.29 (1.59–3.29) for individuals with 2 HRBs (high ST and low PA). Results were very similar for non-infectious disease mortality. As regard to mortality from infectious diseases, results were mostly in the same direction although they were fairly imprecise, and did not achieve statistical significance.

**Table 3:** Mortality risk for inflammatory causes according to groups of Health Risk Behaviors (HRBs).

	**Health risk behaviors**			
	**0 HRB (high PA and low ST)**	**1 HRB (high PA and high ST)**	**1 HRB (low PA and low ST)**	**2 HRB (low PA and high ST)**
*N*	2,447	310	538	382
**INFLAMMATORY CAUSES (*****n*** = **286)**				
Deaths	145	31	61	49
Model 1, HR (95% CI)	1.00 (Ref.)	1.50 (1.00–2.27)	1.78 (1.27–2.49)	2.46 (1.70–3.54)
Model 2, HR (95% CI)	1.00 (Ref.)	1.45 (0.97–2.18)	1.68 (1.19–2.37)	2.29 (1.59–3.29)
**INFECTIOUS INFLAMMATORY CAUSES (*****n*** = **77)**				
Deaths	43	7	15	12
Model 1, HR (95% CI)	1.00 (Ref.)	0.92 (0.40–2.12)	1.22 (0.65–2.28)	1.68 (0.88–3.19)
Model 2, HR (95% CI)	1.00 (Ref.)	0.93 (0.40–2.17)	1.17 (0.62–2.22)	1.67 (0.89–3.15)
**NON-INFECTIOUS INFLAMMATORY CAUSES (*****n*** = **209)**				
Deaths	102	24	46	37
Model 1, HR (95% CI)	1.00 (Ref.)	1.78 (1.11–2.85)	2.05 (1.38–3.04)	2.84 (1.83–4.39)
Model 2, HR (95% CI)	1.00 (Ref.)	1.65 (1.03–2.65)	1.88 (1.25–2.83)	2.55 (1.66–3.93)

*HR, Hazard ratio; CI, Confidence Interval; ST, Sitting Time; PA, Physical Activity; HRB, Health Risk Behavior. High ST was considered 7 h or more of sitting time. Low PA was considered as inactive or less active. Model 1 was adjusted for age (years) and sex (male/female). Model 2 was adjusted for age (years), sex (male/female), educational level (no formal studies/Primary studies/Secondary or higher studies), smoking (never smoker/ former smoker/current smoker), alcohol consumption (never drinker/former drinker/moderate drinker/heavy drinker), body mass index (kg/m^2^), chronic lung disease (no/yes), cardiovascular disease (no/yes), diabetes mellitus (no/yes), Parkinson (no/yes), and cancer (no/yes)*.

Results remained virtually identical after excluding deaths in the first two years of follow-up (*n* = 31 for all non-CVD/cancer inflammatory causes); for example, the adjusted HRs (95%CI) of mortality for all inflammatory causes in those with 1 HRB (low PA) and 2 HRBs were respectively 1.66 (1.16–2.37) and 2.08 (1.39–3.12).

## Discussion

In this prospective study with older adults from Spain we found dose-response associations of PA and ST with mortality from inflammatory causes other than CVD and cancer. Specifically, participants who were moderately active or very active had lower mortality risk compared to individuals who were inactive/less active, regardless of the amount of ST. Additionally, mortality risk was higher among participants who reported high ST (≥7 h/d) compared with participants having lower levels of ST, independently of PA. Results were in the same direction for infectious and non-infectious diseases, though associations were stronger and achieved statistical significance only for non-infectious diseases.

A proinflammatory state, also called “*inflamm-aging*,” is associated with aging and may contribute to many disabling diseases (Sallam and Laher, 2016). However, although inflammatory cytokines increase with age, chronic inflammation might not be necessarily a manifestation of aging “*per se*” and some lifestyle factors, such as PA or ST, may play a prominent role. In this sense, in older adults, regular PA has been systematically linked to reductions in inflammatory biomarkers, including IL-6, TNF-α, and C-reactive protein (CRP) (Elosua et al., 2005), as well as to increased levels of anti-inflammatory cytokines, such as IL-10 (Jankord and Jemiolo, 2004). Prolonged sedentary behavior has also been associated with higher pro-inflammatory markers, independently of PA (Healy et al., 2011; Gennuso et al., 2013; Henson et al., 2013). Due to the effect of PA and ST on chronic inflammation in older people, it is important to assess the extent of the association between these behaviors and mortality from diseases where inflammation plays an important pathogenic role.

The death rates from inflammation-related diseases others than CVD and cancer are substantial; for example, chronic lower respiratory diseases, Alzheimer's disease, diabetes, and kidney disease are among the 10 leading causes of death in the United States (Xu et al., 2016). Similarly, data from the National Institute of Statistics in Spain (www.ine.es) showed that around 20% of deaths in 2015 among adults over 60 years were due to diabetes, Alzheimer's disease or chronic lower respiratory diseases. This highlights the relevance of exploring the modifiable factors associated with these relevant cause-specific mortalities. To the best of our knowledge, this is the first study to examine the independent and combined association of PA and ST with mortality attributed to all inflammatory causes other than CVD or cancer.

Our results are consistent with those of previous studies on the effect of PA on mortality from some inflammation-related diseases. In the Danish Diet, Cancer and Health cohort, participating in sports, cycling, and gardening was linked to reduced risk of death from diabetes (ranged 39–66%) and respiratory diseases in older adults (ranged 37–40%) (Andersen et al., 2015). Moreover, Rosness et al. (2014) reported that, compared with inactive older individuals, the risk of dementia-related mortality was lower in those who engaged in light or vigorous PA during three or more hours per week. Regarding sedentary behavior, Cucino and Sonnenberg (2001) found that mortality related with inflammatory bowel disease was higher in individuals with sedentary vs. manual occupations. Evidence is slightly greater for TV viewing, the most prevalent sedentary behavior in older people (Keadle et al., 2015; Ukawa et al., 2015). Keadle et al. (2015) identified that each 2 h/d increase in TV viewing was associated with an elevated risk of mortality for chronic obstructive pulmonary disease, diabetes, influenza/pneumonia, Parkinson's, and liver disease. In our study, we found that the association between ST and mortality from inflammatory diseases was not linear and the risk of mortality increased when spending ≥7 h/d. Non-linear relationships have also been observed in previous studies examining all-cause deaths; Lee (2016) described that all-cause mortality risk increased linearly when daily sedentary behaviors exceeded 9 h/d. A meta-analysis by Chau et al. (2013) found that the best fitted spline model to characterize the dose-response relationship between sedentary behavior and all-cause mortality was with knots at >3 and >7 h/d.

We also attempted to evaluate the combined effect of PA and ST on inflammatory mortality. When compared to participants who had 0 HRB (high PA and low ST), the mortality risk for inflammatory causes, especially non-infectious causes, was higher in those who presented 1 HRB (i.e., low PA and low ST), and was further increased in individuals with 2 HRB (low PA and high ST). These are in line with earlier research showing the greater protection against morbidity when adequate levels of several lifestyle factors are combined (Sotos-Prieto et al., 2016; Edwards and Loprinzi, 2018). Specifically, our results suggest that interventions addressing concurrently an increase in PA and decrease in ST in older people might have a greater effect on inflammatory survival than those only targeting one of them.

Finally, it is relevant to note that PA and ST were weakly related with mortality attributed to infectious inflammatory causes, so that most associations did not reach statistical significance. The absence of associations may be due to lack of statistical power as the number of deaths related with infectious inflammatory diseases was low (*n* = 77, 26.9% of the total). On the other hand, it is possible that the protective effect of high PA and low ST on inflammation is insufficient to combat external pathogens, particularly in the elderly because of their reduced immune function and greater vulnerability to multiple infections (Bartlett et al., 2016). In addition, the effects of PA on the immune system could vary with the type, intensity and context of PA (Grosset-Janin et al., 2012). A retrospective study with older participants found no relation between overall PA and the number of upper respiratory tract infections (URTI) episodes, but sports participation was negatively correlated with number of URTI episodes during a 1-year period (Kostka et al., 2000). Unfortunately, information on the type and intensity of PA was not available in our study and future research should address this issue.

Our study has some limitations. First, PA and ST were self-reported, and recall biases might have occurred. No data are available on the reliability and validity of the questions utilized in this study, but similar self-report measures have demonstrated adequate validity and reliability in older adults (Van Cauwenberg et al., 2014). Furthermore, information about diet was not collected, and data on PA and ST was ascertained only at baseline so changes in the levels of these behaviors during the follow-up period may have affected the associations we examined. Lastly, although similar classifications of inflammatory diseases have been used previously (Andersen et al., 2006), we acknowledge that they are somewhat subjective.

## Conclusions

Previous research has focused on the effect of PA and ST on CVD and cancer mortality (Nocon et al., 2008; Katzmarzyk et al., 2009; Wilmot et al., 2012), but the effect of these lifestyle factors on mortality from other diseases with a major inflammatory component in older adults has been underexplored. In our study, PA and ST were independently associated with inflammatory mortality other than CVD or cancer in older adults. Also, the highest mortality risk for this cause was observed among older adults with low PA and high ST. Future public health recommendations and clinical interventions should note that addressing both behaviors could have greater benefits on inflammatory mortality than focusing on only one of them. However, our results should be confirmed using objective assessments of PA and ST.

## Author contributions

PG-C, EG-E, and FR-A study concept and design. PG-C, EG-E, and FR-A acquisition of data. VC-S, SH-F, and DM-G analysis and interpretation of data. VC-S and DM-G drafting of the manuscript. VC-S, PG-C, SH-F, EG-E, FR-A, and DM-G critical revision of the manuscript for important intellectual content. VC-S, PG-C, SH-F, EG-E, FR-A, and DM-G final approval of the version to be published. VC-S, PG-C, SH-F, EG-E, FR-A, and DM-G agreement to be accountable for all aspects of the work.

### Conflict of interest statement

The authors declare that the research was conducted in the absence of any commercial or financial relationships that could be construed as a potential conflict of interest.
