# Supplementary material for: Physical Activity, Sitting Time, and Mortality From Inflammatory Diseases in Older Adults
Source: Front Physiol. 2018 Jul 12;9:898. doi: 10.3389/fphys.2018.00898 (PMC6052124; doi:10.3389/fphys.2018.00898)
Supplement: Supplementary file 1 [file Table_1.DOCX]

**Supplementary Table 1.** Number and percentage of deaths for non-CVD/cancer inflammatory diseases (infectious and non-infectious) in the study sample

|  | ICD-10 code | Disease | N (%) |
| --- | --- | --- | --- |
| Infectious inflammatory diseases | | | 77 (26.9) |
|  | A04.7 | Enterocolitis due to Clostridium difficile | 1 (0.3) |
|  | A41.9 | Sepsis, unspecified | 20 (7.0) |
|  | B18.2 | Chronic viral hepatitis C | 5 (1.7) |
|  | J18 | Pneumonia, organism unspecified | 4 (1.4) |
|  | J18.0 | Bronchopneumonia, unspecified | 1 (0.3) |
|  | J18.1 | Lobar pneumonia, unspecified | 9 (3.1) |
|  | J18.9 | Pneumonia, unspecified | 31 (10.8) |
|  | J22 | Unspecified acute lower respiratory infection | 5 (1.7) |
|  | N39.0 | Urinary tract infection, site not specified | 1 (0.3) |
| Non-infectious inflammatory diseases | | | 209 (73.1) |
|  | E11.9 | Non-insulin-dependent diabetes mellitus without complications | 2 (0.7) |
|  | E14.5 | Unspecified diabetes mellitus with peripheral circulatory complications | 3 (1.0) |
|  | E14.7 | Unspecified diabetes mellitus with multiple complications | 15 (5.2) |
|  | E14.9 | Unspecified diabetes mellitus without complications | 9 (3.1) |
|  | E85.4 | Organ-limited amyloidosis | 1 (0.3) |
|  | G20 | Parkinson disease | 16 (5.6) |
|  | G30.1 | Alzheimer disease with late onset | 11 (3.8) |
|  | G30.9 | Alzheimer disease, unspecified | 42 (14.7) |
|  | J40 | Bronchitis, not specified as acute or chronic | 2 (0.7) |
|  | J42 | Unspecified chronic bronchitis | 2 (0.7) |
|  | J43.9 | Emphysema, unspecified | 1 (0.3) |
|  | J44 | Other chronic obstructive pulmonary disease | 12 (4.2) |
|  | J44.1 | Chronic obstructive pulmonary disease with acute exacerbation, unspecified | 11 (3.8) |
|  | J44.8 | Other specified chronic obstructive pulmonary disease | 1 (0.3) |
|  | J44.9 | Chronic obstructive pulmonary disease, unspecified | 27 (9.4) |
|  | J45.9 | Asthma, unspecified | 1 (0.3) |
|  | J47 | Bronchiectasis | 1 (0.3) |
|  | J69 | Pneumonitis due to solids and liquids | 9 (3.1) |
|  | J69.0 | Pneumonitis due to food and vomit | 1 (0.3) |
|  | J84.1 | Other interstitial pulmonary diseases with fibrosis | 5 (1.7) |
|  | K74.6 | Other and unspecified cirrhosis of liver | 6 (2.1) |
|  | K81.9 | Cholecystitis, unspecified | 2 (0.7) |
|  | N12 | Tubulo-interstitial nephritis, not specified as acute or chronic | 2 (0.7) |
|  | N18.9 | Chronic kidney disease, unspecified | 13 (4.5) |
|  | N19 | Unspecified kidney failure | 13 (4.5) |
|  | N32.1 | Vesicointestinal fistula | 1 (0.3) |
